# Factors influencing participation in home, school, and community settings by 6- to 9-year-old children born preterm: a qualitative descriptive study

**DOI:** 10.1007/s11136-025-03993-0

**Published:** 2025-05-23

**Authors:** Tiffany K. Bradshaw, James T. D. Gibbons, Andrew C. Wilson, Amber Bates, Shannon J. Simpson, Jenny Downs

**Affiliations:** 1 Wal-Yan Respiratory Research Centre, The Kids Research Institute Australia, Perth, WA Australia; 2 Curtin School of Allied Health, Faculty of Health Sciences, Curtin University, Perth, WA Australia; 3 Perth Children’s Hospital, Perth, WA Australia; 4 Child Disability, The Kids Research Institute Australia, Perth, WA Australia; 5 Centre for Child Health Research, University of Western Australia, Perth, WA Australia

**Keywords:** Preterm, Middle Childhood, Functioning, Participation, Development

## Abstract

**Purpose:**

There is no published information on preterm children’s activities and participation during middle childhood, a time when growth and development are characterised by increasing motor, reasoning, self-regulation, social and executive functioning skills. This study explored the health, activities and participation of children born very preterm during middle childhood (6–9 years) from the perspectives of their parents.

**Methods:**

This was a qualitative descriptive design. Twenty parents of 27 very preterm children born < 32 weeks gestation participated in semi-structured interviews to explore their child’s health, behaviour, functioning and participation in school, at home and in the community along with environment and personal factors that influenced the child’s activities and participation. Interview data were coded to each of the International Classification of Functioning in Disability (ICF-CY) domains.

**Results:**

Parents reported a broad range of health needs and participation outcomes. Parents reported challenges related to respiratory health, mental health and behaviour, sleep, nutrition and feeding. The child’s participation and functioning were influenced by both personal and environmental factors including but not limited to parenting styles, education and learning support, access to health support and personal preferences and motivations.

**Conclusion:**

Preterm birth is associated with impacts on the child’s health, activities and participation. This comprehensive view on the child’s health and wellbeing can aid clinicians in their management of these children.

## Plain English summary

Many people who are born too early (or preterm) are at an increased risk of poor physical health across their life with early onset of chronic illness and neurological, mental health or behavioural challenges. However, health is not just the presence or absence of disease, it is defined broadly as wellbeing across physical, mental and social domains. Parents want to know how health impacts how their child lives.

In this study, parents of children born preterm participated in interviews over the phone or via video-call to talk about their child’s health, sleep, behaviour, daily activities and participation in school, at home and in the community.

Parents described a very broad range of health needs and participation outcomes in their child.

This study found that persistent health needs for children born preterm are common in middle childhood, with many children born preterm having ongoing developmental needs. Ongoing health needs appear to interact with capacity for activities and participation.

This study is the first to ask parents about how the health, environmental and personal factors impact the child’s daily activities and participation when they reach middle childhood. This study will help to inform future guidelines on the need for a multi-disciplinary care approach in the clinical follow-up of these children.

## Introduction

An estimated 13·4 million babies were born preterm (< 37 weeks gestation) globally in 2020 equating to approximately 10% of livebirths [1]. Surviving preterm birth is associated with increased risk of poor physical health across the lifespan and early onset of chronic diseases spanning multiple organ systems. Many individuals born preterm have impairments in lung health [2], including increasing airway obstruction over time [2], and increased rates of asthma [3] and chronic obstructive pulmonary disease [4]. Sleep is impacted, with preterm toddlers having more night-time wakings and poorer sleep efficiency than their term-born peers [5], and 5–12-year-old preterm children sleeping approximately one hour less than population recommendations for age [6]. Cardiovascular, renal and endocrine systems are all at increased risk of chronic dysfunction in those born preterm [7]. In a meta-analysis of 64,061 children, lower scores across cognitive, motor, behaviour and academic domains were reported for preterm children compared with their term-born peers [8]. The odds of being diagnosed with ADHD [9] and risk of developing autism [10] and anxiety [11] are higher in preterm children compared with their term-born peers. Earlier gestational age at birth and lower birth weight is associated with a higher risk of developmental impairments [8, 9].

Not surprisingly, there has been enormous focus on the neonatal and early childhood periods for children who were born preterm because of the risks that preterm birth imposes on development and critical body systems such as the cardiorespiratory and neurological systems [12]. Since the 1970s, preterm birth survival rates are increasing [13] and risks of adverse outcomes are decreasing. For example, the birth prevalence of cerebral palsy in Australia declined markedly between 1995 and 2016 [14]. Follow-up research now extends into adulthood [12]. However, health is broadly defined as wellbeing across physical, mental and social health, and does not simply equate to the absence of disease impairments [15]. Parents are acutely interested in functional aspects of their child’s health [16]. As documented in the International Classification of Functioning in Disability (ICF-CY), wider evaluations of how children live, including their day-to-day activities and participation, along with influencing personal and environmental factors, can provide important insights into how a child lives with disease or impairments [17].

For children born preterm, less is known about the middle childhood period (age 6–11 years) [18]. Typically, children are continuing to develop motor, reasoning, self-regulation, social and executive functioning skills, whilst attending school, making friends and engaging with activities in the community [18]. The ICF-CY conceptualises the inter-relationships between health, activities, participation and the environment which together are critical determinants of quality-of-life [19].

To our knowledge, there is no published information on preterm children’s activities and participation during middle childhood. The aim of this study was to explore how very preterm (< 32 weeks gestation) children in middle childhood functioned in their day-to-day lives. Guided by the ICF-CY, we explored their health, activities and participation, and environmental and personal factors, from the perspectives of their parents.

## Methods

### Study design

This was a descriptive, qualitative study that sought to explore the health, activities and participation of children aged 6–9 years who had been born very preterm. Ethical approval was obtained from the Child and Adolescent Health Services Human Research Ethics Committee (RGS5034) and informed consent was obtained from parents.

### Participants

Parents of children born < 32 weeks gestation were recruited to the Preterm Infant Functional and Clinical Outcome (PIFCO) study (HREC Reference: 2013091EW) while their baby was in the neonatal intensive care unit at King Edward Memorial Hospital, Perth, Western Australia. The children (n = 293) of parent participants were born 2013 to 2017 and families have since been invited to participate in ongoing respiratory follow-up studies. For this study, children with no severe congenital abnormalities, cardiopulmonary defects or severe neurodevelopmental impairment that prevented them from completing lung function measurements were recruited as part of a mid-childhood respiratory follow-up study, which was approved by the Western Australian Child and Adolescent Health Service Human Research Ethics Committee (RGS5034) on July 4 th, 2022. During ongoing recruitment for this respiratory follow-up, all parents of these children were invited to participate in this current sub-study. With their written consent, parents were recruited to the current qualitative study using a purposive sampling strategy to capture a range of child gestational age, biological sex, area of residence (metropolitan, rural) and single and multi-pregnancies. Multiple births are common in preterm cohorts because multiple pregnancy is a risk factor for preterm birth [20], such that approximately 20% of preterm births are also a multiple birth [21]. We estimated that we needed an approximate sample size of 20 interviews to achieve information power [22]. Information power is a concept suggesting that sample sizes in qualitative research should be considered in terms of the research aims, methods, focus, and data quality (i.e., the perceived wealth of information held by the sample) [22].

### Procedures

The development of this study involved working closely with our Preterm Consumer Reference Group (CRG). The Preterm CRG meets formally 4 times a year along with calls for occasional out of session consultations. The Preterm CRG comprises 15 adult members, including some who were born preterm and parents (mothers & fathers) of preterm children. The preterm children range from infancy to young adulthood. Members reside in both metropolitan and regional areas, with many having been involved or had children involved in previous preterm research studies. A series of open-ended and probing questions were reviewed and modified with the Preterm CRG. They provided valuable insight from their own lived experiences on the aims of the study, content of the interview schedule, why the questions were important and ensured that we were including a suitable spread of families.

In the interview schedule, we first queried the child’s health, behaviour, medical diagnoses, treatments and what the child enjoyed doing. Second, open-ended questions explored the child’s activities, participation, and any influencing factors (e.g., *“Tell me about the things your child does at school [classroom activities, playground activities, after-school activities]. Do you think your child’s health impacts on their ability to do these things? If so, how?”)*. Probing questions sought examples of relevant scenarios, for example: *“Can you give me some examples?”* or *“What would I see if I was there watching?”*. Parents were asked about their child’s strengths and challenges.

Training was provided by the senior author (JD) to the primary researcher (TB). Prior to the interview, parents were sent the interview schedule enabling them to prepare for the interview if desired. Interviews were conducted via video call or telephone, depending on parental preference. Consent to record the interview was obtained at the start of the interview.

Perinatal data were extracted from data collected at initial recruitment to the PIFCO study. Quality of life was assessed using KIDSCREEN-27, a validated 27-item measure of child health-related quality of life [23], used previously in a preterm population [24]. Domains include physical well-being, psychological well-being, autonomy and parents, social support and peers, and the school environment [23]. A modified International Study of Asthma and Allergies in Childhood (ISAAC) questionnaire, validated for use in paediatric populations as a standardized assessment of asthma and respiratory symptoms along with other allergic disease [25] was also completed. Parents completed both questionnaires via a survey link after the interview or in person at their child’s research appointment for respiratory follow-up.

### Data management and analysis

Interviews were transcribed and checked for accuracy by simultaneously listening to the interview recording and reading the transcript. A copy of the transcription was provided to the parents for checking to clarify statements and to offer more information if they chose to do so. Transcripts were re-read multiple times to identify key themes in the interviews. Data were coded in NVivo using content analysis [26]. First, one investigator (TB) used open coding to understand potential categories and develop a coding framework. This was discussed by two investigators and intermittently reviewed (TB, JD). The final coding framework represented each of the ICF-CY components and analysis was deductive. When approximately half of the interviews had been coded, the coding framework was presented to the CRG for their feedback. The CRG suggested additional review of the data to search for codes describing nutrition and eating.

### Trustworthiness

To ensure trustworthiness, stringent guidelines were followed during data collection and analysis to maintain credibility, transferability, dependability, and confirmability throughout the study [27]. Review and discussion of findings with the CRG supported the credibility of the analysis, ensuring that our data and analyses was consistent with their experiences. Regular peer checking and debriefing was used to limit investigator bias with joint development of the coding framework. Purposive sampling was used to enable transferability of the data. We held regular investigator meetings and developed a detailed record of the data collection process to enhance dependability and confirmability.

## Results

### Demographics

Twenty parents of 27 very preterm children were interviewed from a recruitment pool of 60 children. Only 1 child’s parent declined consent to participate in the interview. We estimated that thematic saturation was achieved as the final 3 interviews did not yield information for new categories. Phone or videocall interviews (n = 19 mothers, n = 1 father) were conducted ranging from 29 to 79 (median 52) minutes in duration. Slightly more than one third (7 interviews) of families lived in rural locations. Of the 20 interviews, 5 were parents with twins and 1 with triplets. The children had a median gestational age of 28.6 (range 24.0–31.7) weeks and were a median of 7.8 (range 6.5–9.9) years at the time of interview. Having received a diagnosis of ADHD was reported by parents in almost 19% of the cohort, autism and asthma in 11%, and dyslexia in 7%. Respiratory symptoms were described for more than half of children in the past 3 months, with 59% of parents reporting wheeze, cough, rattle and/or shortness of breath in their child (Table 1). Mean parent-reported KIDSCREEN-27 domain T-scores ranged from 51.7 for the Parent Relations and Autonomy domain to 57.0 for the Physical Wellbeing domain. The sample characteristics are presented in Table 1.

**Table 1:** Characteristics of the 27 very preterm children, born to the 20 parents who participated in an interview

Demographic characteristics	Gestational age, weeks (median, range)	28.6 (24.0, 31.7)
	Birth weight, z-score (mean, SD)	0.11 ± 0.73
	Age, years (median, range)	7.8 (6.5, 9.9)
	Male, n (%)	15 (55.6%)
	Rural, n (%)	10 (37.0%)
	Multiple Pregnancy, n (%)	13 (48.1%)
	Ethnicity, n (%) Indigenous Caucasian Other	4 (14.8%) 17 (62.9%) 6 (22.2%)
Neurodevelopmental co-morbidities	Autism, n (%)	3 (11.1%)
	ADHD^ (clinical diagnosis), n (%)	5 (18.5%)
	Undergoing diagnosis testing for ADHD, n (%)	2 (7.4%)
	Dyslexia, n (%)	2 (7.4%)
Respiratory co-morbidities	Bronchopulmonary dysplasia*, n (%)	12 (44.4%)
	Asthma ever, n (%)	3 (11.1%)
	Respiratory symptoms in the last 3 months, n (%)	
	Wheeze	6/27 (22.2%)
	Cough	16/27 (59.3%)
	Rattle	4/25 (16.0%)
	Shortness of Breath	4/25 (16.0%)
	Impacts of respiratory symptoms over the previous 3 months, n (%)	
	Affected their eating	6/27 (22.2%)
	Woke them up	9/27 (33.3%)
	Reduced their activity	9/26 (34.6%)
	Occurred during exercise	12/27 (44.4%)
	Made them unusually tired	7/25 (28.0%)
	Limited family activities	6/27 22.2%)
	Disturbed family sleep	9/26 (34.6%)
	Respiratory hospital admission ever, n (%)	9/24 (37.5%)
	Age of first respiratory admission, months (median, range)	8.9 (1.67, 43.3)
KIDSCREEN-27 domains	Physical Well-being (n = 27; mean, SD), t-value Psychological Well-being (n = 27; mean, SD), t-value Parent Relations & Autonomy (n = 24; mean, SD), t-value Social Support & Peers (n = 27; mean, SD), t-value School Environment (n = 27; mean, SD), t-value	57.0 ± 10.4 52.6 ± 8.5 51.7 ± 7.3 54.6 ± 9.7 56.1 ± 12.4

^*^BPD Bronchopulmonary dysplasia, (classified having BPD if received 28 days of oxygen supplementation or more, as assessed at 36 weeks postmenstrual age)

^*ADHD* Attention-deficit/hyperactivity disorder

### Health, activity and participation

**Fig. 1:**
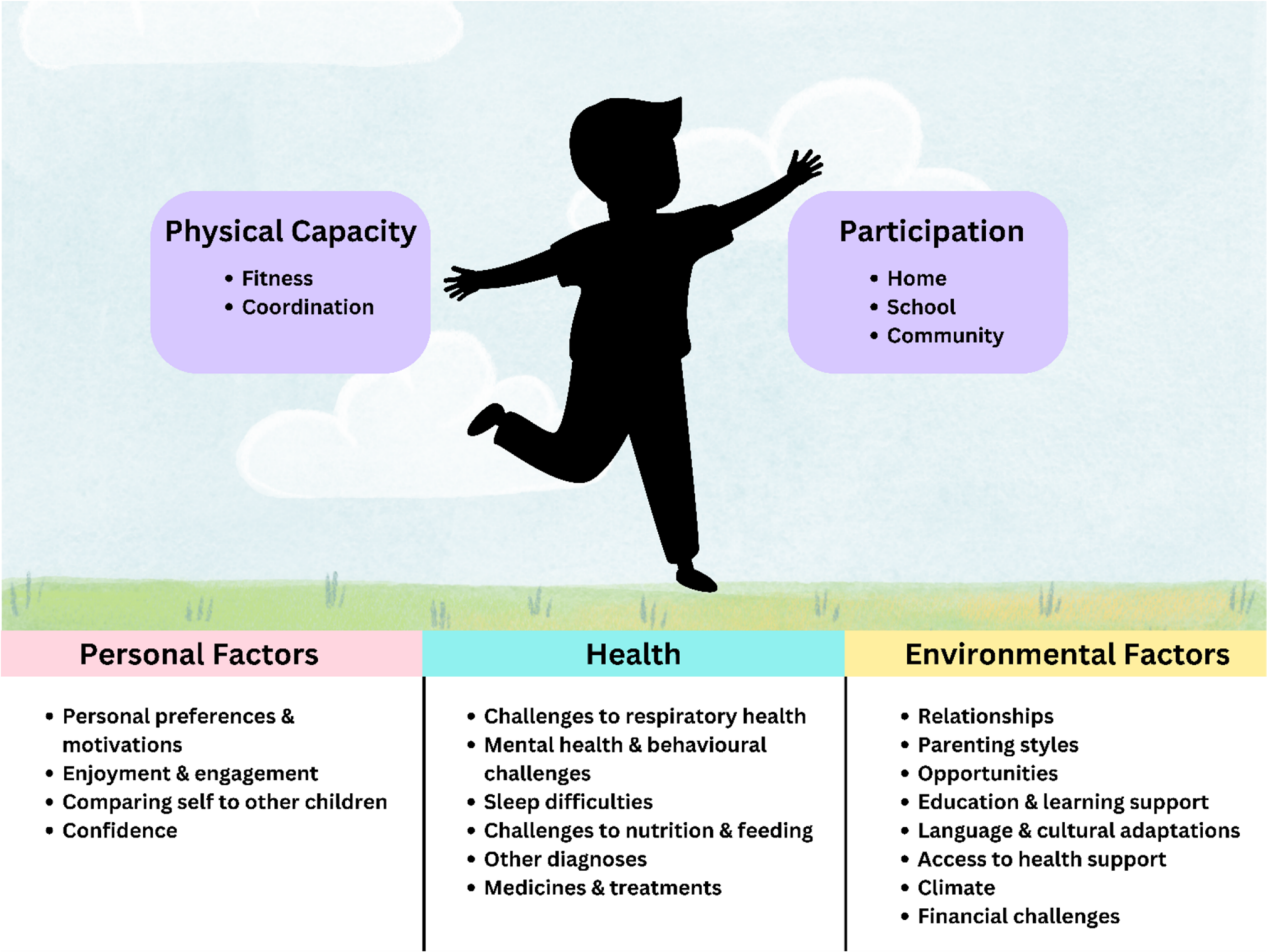
Illustration of the health, personal and environmental factors that underpinned activities and participation in children who had been born very preterm

Health, personal and environmental factors were described as influencing the child’s activities and participation, as summarised in Fig. 1. Each ICF-CY category was characterised by strengths and challenges indicating a broad range of health needs and functional and participation outcomes. The categories are summarised with example quotes below. Categories and codes for health are presented in Table 2, activities and participation in Table 3, and environmental and personal factors in Table 4. Pseudonyms are used for all quotations.

**Table 2:** Categories in the Health domain with example codes illustrating strengths (S) and challenges (C)

Category	Example codes	
*Respiratory Health*	S:	Good respiratory health Respiratory health improving with growth
	C:	Susceptible to picking up colds and flus Recurrent symptoms such as cough Long recovery times Tight chest or breathing difficulties when exercising Asthma and allergies
*Other Diagnosis*	S:	Good health
	C:	Other diagnoses such as Autism, ADHD, Nephrocalcinosis, Dyslexia Ongoing problems with Low weight, Enuresis, Constipation
*Medicines & Treatments*	S:	No medications or treatments
	C:	Medications to manage respiratory symptoms, poor sleep, ADHD symptoms Schedule of regular medical and/or specialist appointments
*Mental Health & Behaviour*	S:	Good mental health and regulated behaviours
	C:	Anxiety Challenges to mood Challenges to thinking and problem solving Emotional dysregulation, Behavioural challenges, Hyperactivity & impulsiveness
*Sleep*	S:	Sleeps well at night
	C:	Struggles to fall asleep and requires support to get to sleep Wakes up tired Snores Co-sleeps with parent(s)
*Nutrition & Feeding*	S:	Eating adequate quantity and variety of foods
	C:	Reduced appetite Challenges to weight gain Challenges to eating Specialised diet

**Table 3:** Categories in the Activities and Participation domains with example codes illustrating strengths (S) and challenges (C)

Domain	Category	Example codes	
Activities	*Physical capacity*	S:	Keeps up well with other children Improvements in physical capacity with growth
		C:	Difficulties with coordination, poor muscle strength and low muscle tone Impacts from mental health & behavioural challenges Struggles to keep up with peers Tires and fatigues easily, poor stamina and endurance
Participation	*Home*	S:	Independent self-care Participates in household chores & domestic activities Finds downtime and entertainment activities inside the house Participates in active activities in the outdoors
		C:	Requires prompting to do self-care tasks and poor concentration impacts task completion Requires prompting and extra encouragement to help with domestic tasks Procrastination getting started with tasks
	*School*	S:	Has friends, Gets along with peers Making progress, keeps up with their school level, excels in some areas of schooling Concentrates and stays on task Participates in afterschool care activities
		C:	Poor concentration, struggles academically, has learning difficulties Absence from school due to poor health, participation in school affected by sub-optimal health Easily fatigues Anxiety impacts going to school and task completion at school Struggles with social and emotional skills Treated differently to peers
	*Community*	S:	Participates in sports and recreational activities, e.g., takes music, art, or dance lessons Participates well in unstructured activities Gets on well with other children and makes friends easily
		C:	Lack of confidence or mental challenges or fatigue impact participation Struggles with concentration and following instructions Struggles to keep up with peers Poor sleep & bed-wetting prevent participation in sleep overs

**Table 4:** Categories of Environmental and Personal factors that influenced the child’s participation

Domain	Category	Example codes
Personal factors	*Home, school & community*	Preferences and motivations Confidence Enjoyment and engagement Comparing self to other children
Environmental factors	*Home*	Sibling relationships or being an only child Has pets Opportunities of inside downtime activities Parent relationships and parent child relations Parenting styles, e.g., monitoring screen time Own or shared bedroom Health conditions of other family members Financial challenges
	*School*	Peer relationships Education and learning support, supportive school environment English not the first language spoken at home and cultural adaptations
	*Immediate Community*	Opportunities for a variety of out of school recreation and arts activities, e.g., may be limited in rural settings Involved in church
	*Broader environmental factors*	Relationship with General Practitioner Access to necessary specialist and/or allied health appointments Climate may restrict activities, e.g., excessive heat

#### Strengths and challenges to health

Discussions with parents focussed on respiratory and sleep health, developmental and behavioural challenges, and mental health (Table 2). Some children had ongoing issues with low body weight, poor appetite, enuresis*,* and constipation (Table 2).

Substantial improvements in respiratory health as the child grew older were described. “*When he was younger, whenever he got an infection, it would go straight to his chest. And up until he was about three, it was usual for a cold to result in having to go to hospital just to monitor his breathing … but that kind of phased out … He is very rarely unwell.” (Laura)* For others, increased susceptibility to colds and flu with recurrent respiratory symptoms persisted. “*And even now, when she gets sick, she gets sick really quick, really, really bad.” (Jane).*

Approximately half the participants reported sleep difficulties, including children struggling to fall asleep, waking up tired, snoring, or needing to co-sleep. For some, challenges with sleep had marked impacts on the child’s daytime behaviour. “*We get six nights of [poor] sleep and then that one night where he has his melatonin and he gets a good night’s sleep, … the next day, there’s no fighting, does what I need him to do, happily gets up in the morning… And then the nights when he doesn’t and he’s grumpy, he’s a little cyclone.” (Claire).*

Many parents described good mental health and behavioural regulation in their children. For others, the child experienced challenges with anxiety, mood, their ability to concentrate and problem solve, regulate their emotions, and hyperactivity and impulsiveness. For example, *“-she’d probably have a meltdown once every couple of days…. Crying, screaming, yelling, stomping her feet, flapping her hands, dropping to the ground.” (Ruby)* A few children were reported to have received a diagnosis of autism and/or ADHD, *“With their prematurity, they have had some challenges with development delays including moderate autism and ADHD.” (Heidi).*

Some children had developmental delays that impacted their academic work, or they were seen as ‘younger’ by their peers, *“I would say she’s at least a year to two years’ maybe behind the other kids… I don’t think the other kids view her as being the same age as them. And she doesn’t quite have the same level of maturity, I suppose, as the other kids.” (Kerry).*

Other children had developmental delays that were well supported by their environment, for example, *“it’s a special needs school for children with autism, and they have mostly children with autism or Down syndrome. She also has a speech therapist and OT and a psychologist come to school to do sessions with her at school, where she goes off and does them one-on-one…When she came over [from mainstream schooling], she couldn’t read. She didn’t even smile. There were no facial expressions. She didn’t eat at school, she didn’t use the toilet, nothing.” (Jane).*

#### Activities and participation

##### Physical activities

Regarding physical activities, some parents described their child as keeping up well with their peers, many noting that capacity for physical activity had improved with growth (Table 3). For example, *“The only thing it took him a long time to get was his strength and his physical strength. But now … he’s a bit older; he’s a bit more willing to jump in and get amongst it sport wise.” (Bridget)* Other children experienced difficulties with coordination. “*… she’s not that coordinated. She’s not very strong.” (Grace).*

##### Participation at school

Parents described their child’s participation across academic and social domains (Table 3). For some children, academic participation at school was impacted by poor concentration, learning difficulties, fatigue and absences from school due to poor health. For example, “… *he needed to be on the individual education plan as … he wasn’t going to be concentrating, so he wasn’t going to be learning as much.” (Bridget)* For other children, anxiety impacted going to school and task completion.

Variable relationships with peers and personal motivations for school were described, for example, *“…every morning she’ll wake up and say there’s something wrong, [and say] ‘Can I not go to school today?’—She’d much rather prefer being at home with us, because she does struggle with the work compared to the other kids”. (Kerry)* In comparison, some other parents indicated that their children were very enthusiastic about school, “*[They] Love school. Yeah. Every aspect of school. They’re very social, they love the classroom side of it as well. They leap out of bed in the mornings to go to school.” (Bronwyn).*

##### Participation at home

Most children could independently complete self-care tasks such as brushing their teeth, getting dressed, showering and getting ready for school (Table 3), *“… she has a little jobs chart for getting ready for school, which is mainly just her stuff like get up, get dressed, make your bed, need to brush your teeth, and have breakfast, and all of that…” (Anne)* Many children participated in household chores and domestic activities (Table 3), *“…both girls cleaned their bedrooms, folded their clothes, did the laundry, picked the dog poo up, put the bins out, did the dishwasher, did the washing, cooked dinner, made some cakes and vacuumed the floor.” (Matthew)* Some children procrastinated or their concentration impacted task completion and prompts and encouragement were then needed. At home, many parents described their child enjoying activities in the outdoors and entertainment activities inside the house for downtime (Table 3).

##### Participation in the community

Many children participated in sports and recreational activities such as music or dance lessons (Table 3). Enjoyment of social interactions and developing friendships supported their involvement in sport and recreation activities. For example, *“there’s nine of them that are really, really good friends. They never fall out. … They’re on this netball team. She absolutely loves it. So, they train on a Thursday and play on Friday.” (Elizabeth).*

For other children, participation in community activities was restricted by low confidence, difficulties with concentration, fatigue or anxiety (Table 3). For example, *“She needs me to stay there though, for her to feel confident to do it [art class], so that’s part of her anxiety.” (Grace)* Low confidence also affected communication which was also a barrier to participation. *“No, but she doesn’t [do extracurricular activities] …part of what holds her back there is the inability to communicate with people she’s not confident with. Stuff like that. So, we are giving that a little bit more time…as soon as we go out, the words just stop.” (Jane).*

Poor sleep and bed-wetting prevented some children from participating in sleepovers with their friends. *“… we told them when they stop bedwetting, ‘We will send you.’ Sometimes they feel embarrassed, I know especially when their friends come here for sleepovers, so they just try to not tell them and hide themselves and hide their diapers. Yes, they miss sleepovers.” (Gemma).*

#### Environmental and personal factors influencing participation

Personal factors such as confidence, enjoyment and engagement with specific activities, and sibling and friend relationships, were motivators for the child’s activities and participation (Table 4).

Some personal preferences were tied to the child being aware of their own physical capacity and limits, for example, *“the other week she said, ‘Well, I was on my own at lunch today.’ And I said, ‘Oh, how come?’ And she said, ‘Well, they wanted to play, and they were playing a game that…’ Because she’s not very fast, she didn’t run very fast. So she said, ‘They were playing this game, and I can’t run very fast, so I didn’t want to play.’” (Elizabeth).*

Personal preferences for teachers, along with opportunities to socialise influenced participation in sports, *“She used to really like her sport teacher, so she joined the running club for a year, as she really, really liked her teacher. But then the other teacher started going so she didn’t want to go anymore. And so, does she enjoy running club? The social aspect, yes. But why is she there? Is it because she wants to be a good runner? Not really.”* (*Lisa).*

Building friendships and the influence of other children also motivated participation, *“as he’s gotten a little bit older, and he’s made lots of different friends and some of them are into soccer, some of them into footy, some of them into other things, he’s pushed himself more. And so, then his social groups got a bit bigger, so his friends push him, his peers.”* (*Bridget).*

Activities and participation were strengthened by a supportive environment, at school, at home and in the community (Table 4). For example, access to learning supports enabled greater participation at school. *“… they’ve got breakout areas at the school. So sometimes when they see that they’re a bit tired [their educational assistant will] say, let’s go to your teepee. …why don’t you just have a rest for a little bit, see how you’re feeling, read a book … and then come back to the class.” (Heidi)* For some children, parents made adjustments to account for the child’s fatigue and enable the child to have domestic chores and roles. This included making sure they have a checklist to enable the child to independently get themselves ready for school in the mornings, *“he’s got a checklist and things like he can only watch TV once his bag is packed by the door and his shoes are on. … Get dressed, shoes on, make breakfast, and if it’s toast, we make it for him. If it’s cereal, he can do it. Pack bag, brush teeth, put bag by the front door, and then he can watch TV.”* (*Laura).*

Opportunities for community participation were varied and appeared to be more limited for those children living in rural settings, but options suitable for the child’s preferences could usually be selected (Table 4).

### Greatest strengths and challenges in very preterm children

**Fig. 2:**
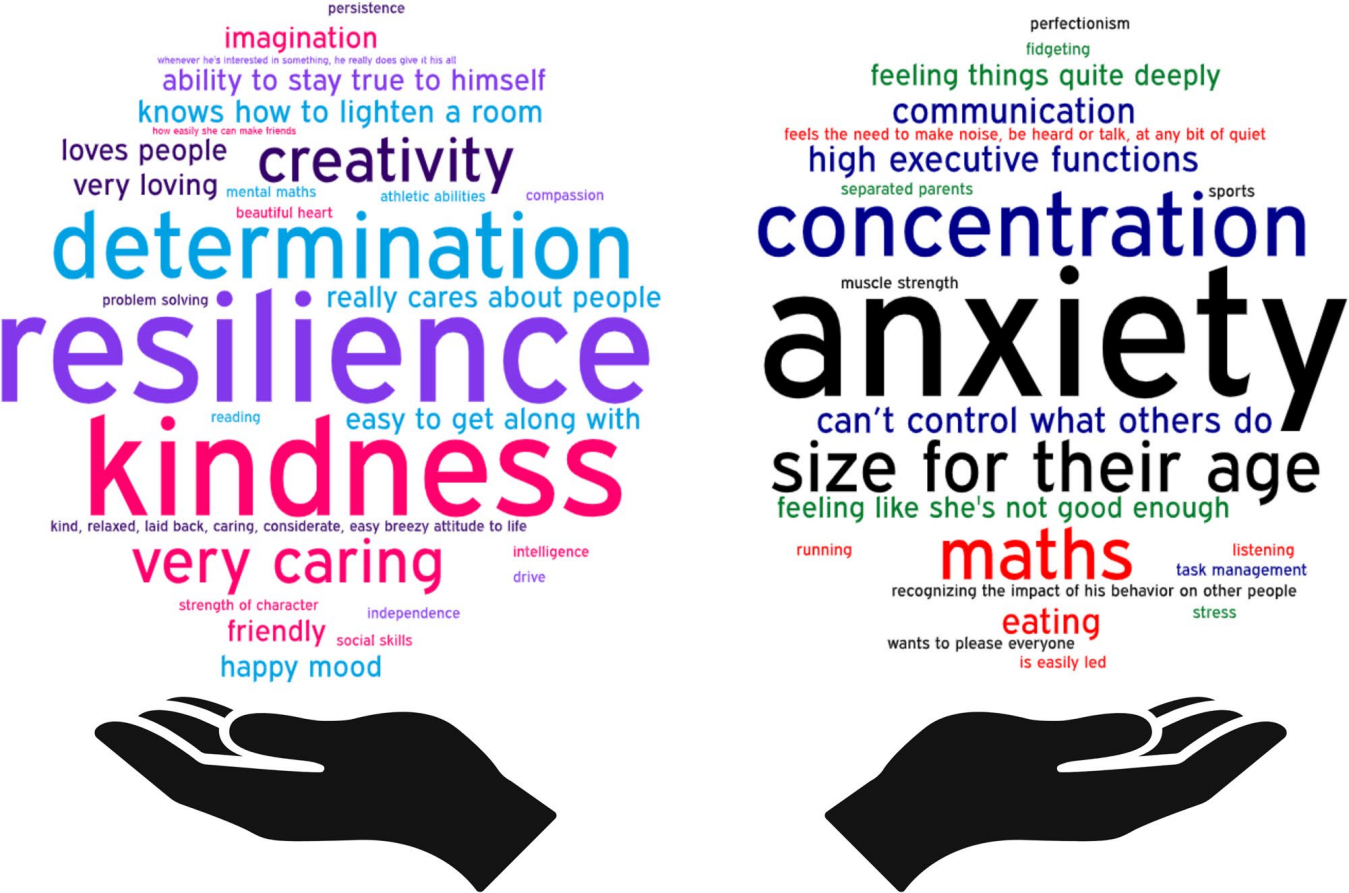
Word cloud presenting parent descriptions of their child’s greatest strengths and challenges. Larger words appeared more frequently in the source text

Parents briefly described their child’s greatest strength(s) and biggest challenge(s) which are presented in word clouds (Fig. 2). Resilience, kindness and determination were frequently described as great strengths in the children. Anxiety and poor concentration were expressed most frequently by parents as the biggest challenges their child faced (Fig. 2).

## Discussion

This study described the impacts of health conditions, environmental factors and personal factors on activities and participation of very-preterm children during middle childhood. Complex and diverse strengths and challenges were identified in these children.

Consistent with the literature [2], some children lived with persistent challenges to their respiratory health, including asthma, other respiratory symptoms and poor sleep. Symptoms of insomnia affect 15–30% of children and adolescents in the general population [28] with marked negative impacts on mental health and quality-of-life [29]. However the prevalence is higher in children with chronic conditions, such as asthma [30] and neurodevelopmental conditions [31]. For preterm-born children, poor sleep including insomnia can persist throughout early and middle childhood [5, 6]. There are critical bi-directional relationships between sleep and physical, mental and social wellbeing [32]. These bi-directional relationships were also illustrated in our data which in-turn appeared related to their participation at school, home and in the community. Other everyday issues reported by parents included gastrointestinal health, growth and nutrition needs. Nutrition needs in middle childhood may receive less attention than during other stages of childhood [33], yet good nutrition in the school-age years, is critical for maintaining adequate growth and neurodevelopment, addressing any “catch-up” that may be necessary following any undernutrition associated with preterm birth, and at the same time avoiding the development of overnutrition [33].

Preterm infants have increased risk of mental health and behavioural problems and neurodevelopmental delay [9], also described in this study as impacting the child’s participation and functioning at school, at home and the community. Being born preterm is a risk factor for anxiety [11]. A meta-analysis reported rates 3.3 times higher rate of diagnosis with ASD in preterm children than in the general population (95% CI 0.24–47.60) [10]. A large cohort study in Finland [9] has shown increased risk of ADHD with each earlier week of gestational age [9]. Behavioural, social and emotional problems in preterm individuals persist into early adulthood, impacting their home life, relationships, school and activities when compared to term born controls [34]. These data underscore the need for a vigilant approach to evaluation and support for neurodevelopmental impairments in the preterm population, including in middle childhood.

Middle childhood is a time when children are actively participating in domestic activities [18] and many parents described their child independently completing self-care tasks and enjoying inside and outside activities in their downtime. In a large population study, there was an inverse relationship between gestational age at birth and prevalence of special education needs [35]. In our smaller sample where no child had been diagnosed with cerebral palsy, there was variability for how physical and mental health factors impacted participation at school. Participation in the community provides opportunities to learn skills and socialise and is a critical determinant of quality-of-life. Again, physical and mental health challenges appeared associated with variable impacts on community participation. We measured quality-of-life with KIDSCREEN-27 [23] and each of the dimension scores were comparable to population norms, consistent with observations in a Swiss cohort study of teenagers who had been born at < 30 weeks [24]. It is possible that personal and environmental strengths enabled the children in our study to participate optimally at home, school and in the community, favourably influencing quality-of-life scores, despite the children’s physical and mental health vulnerabilities.

The long-term healthcare outcomes of children born preterm represent a significant healthcare burden to individuals, families and society. Recent developmental guidelines in Australia recommend structured, preterm-specific follow-up care for children born very preterm [36]. However, the follow-up guidelines focus on the preschool period, meaning some cognitive or behavioural issues that may arise later in development or assessments of respiratory disease that are more feasible during middle childhood may be missed. A European study has described heterogeneity in eligibility criteria, duration and content in preterm follow-up programs across 11 countries, the majority to 5 years but with programs in the Netherlands and Portugal in place to 8 years [37]. Our exploratory data showed that the sample experienced a heterogeneous set of physical and mental health issues that continued into middle childhood and appeared to interact with the child’s activities and participation, which are critical outcomes for parents [16]. Whether these complex needs would be more comprehensively and efficiently supported in a structured multidisciplinary follow-up clinic setting, beyond the early childhood period, is not known, but its value should be investigated.

### Strengths and limitations

There are several strengths and limitations to this study. Credibility of the findings was supported by consultation with our CRG who provided critical input to the design of our interview schedule, refining the coding framework and interpreting the data. Transferability was enhanced by variation in the participant characteristics of gestational age, sex and the proportion of children who lived rurally. Limitations were that our participants were mostly mothers and only one father were interviewed. There were no children in our study with cerebral palsy although being born preterm is a risk factor for cerebral palsy [35], and accordingly, we have not captured the factors affecting participation for this group. Diagnoses of ADHD, autism, dyslexia and asthma, were all parent reported formal diagnoses.

## Conclusion

This study found that health, personal and environmental factors appeared to influence activities and participation of very preterm children. This study will help to inform future strategies for multi-disciplinary care and clinical follow-up of preterm children in middle childhood.

## Data Availability

The data that support the findings of this study are not openly available due to reasons of sensitivity and are available from the corresponding author upon reasonable request. Individual participant data that underlie the results reported in this article would be available after de-identification, assuming participant consent was given. Data would be made available to investigators providing a sound proposal that has been approved by an independent review committee to achieve the aims of the proposal. Proposals should be directed to shannon.simpson@thekids.org.au; to gain access, data requestors will need to sign a data access agreement.

## Abbreviations

ICF-CY: International Classification of Functioning in Disability for Children and Youth
ADHD: Attention-deficit/hyperactivity disorder
HREC: Human Research Ethics Committee
CRG: Community reference group
ASD: Autism spectrum disorder
